# Smooth borders between inner nuclear layer and outer plexiform layer predict fewer macular edema recurrences in branch retinal vein occlusion

**DOI:** 10.1038/s41598-021-95501-w

**Published:** 2021-08-06

**Authors:** Hirofumi Sasajima, Kotaro Tsuboi, Rokuki Kiyosawa, Akira Fukutomi, Kenta Murotani, Motohiro Kamei

**Affiliations:** 1 Department of Ophthalmology, Shinseikai Toyama Hospital, Imizu, Toyama Japan; 2 Department of Ophthalmology, Aichi Medical University, Nagakute, Aichi Japan; 3 Casey Eye Institute, Oregon Health & Science University, Portland, USA; 4 Biostatistics Center, Graduate School of Medicine, Kurume University, Fukuoka, Japan

**Keywords:** Predictive markers, Retinal diseases

## Abstract

We hypothesized the smoothness of the border between the inner nuclear layer (INL) and outer plexiform layer (OPL) associates with the frequency of macular edema (ME) recurrences secondary to branch retinal vein occlusion (BRVO). Thirty-seven consecutive eyes with BRVO treated with anti-vascular endothelial growth factor (VEGF) injections at 1-year follow-up were included. We manually traced the border between the INL and OPL within the 1.5-mm vertical line from the fovea on optical coherence tomography (OCT) images at the initial visit. The jagged ratio (JR), the border length divided by the spline curve length, was calculated. We performed univariate and multivariate regression analyses, including JR, patient characteristics, number of cystoid spaces in the INL, INL area, and outer retina area. Multivariate regression analysis showed JR significantly correlates with the total number of anti-VEGF injections (*P* < 0.0001). Moreover, the mean JR was significantly lower in the nine eyes receiving two or fewer injections than in the 28 eyes receiving three or more injections (1.02 ± 0.01 vs. 1.13 ± 0.06, *P* < 0.0001). A smooth border between the INL and the OPL on OCT images at the initial visit may be a biomarker for fewer ME recurrences in eyes with BRVO.

## Introduction

Macular edema (ME) is the leading cause of visual loss related to branch retinal vein occlusion (BRVO)^1–3^. In the treatment of ME secondary to BRVO, the efficacy of anti-vascular endothelial growth factor (VEGF) agents has been reported in large randomized trials, with anti-VEGF agents as first-line treatment^4–7^. The anti-VEGF injection regimens in the clinical trials, such as BRAVO and VIBRANT, included 6 monthly injections regardless of ME in the first 6 months^4, 7^. However, whether multiple anti-VEGF injections are necessary to initially treat ME secondary to BRVO remains unclear. Generally, approximately 20–30% of patients with ME secondary to BRVO require a maximum of one to two anti-VEGF injections annually^8, 9^. The ZIPANGU trial, which is a prospective clinical trial that followed a pro re nata (PRN) regimen, reported that almost one-fourth of the eyes treated with ranibizumab (Lucentis, Genentech Inc., South San Francisco, CA) monotherapy require two or fewer injections annually^9^. These results suggest that the frequency of ME recurrence is different among BRVO eyes. Moreover, factors predicting fewer ME recurrences remain unclear. Thus, a good predictor of ME recurrence is needed to select appropriate treatment strategies.

Previous fluorescein angiography (FA) studies demonstrated that the angiographic feature of incomplete capillary loss may be associated with recurrent ME in eyes with BRVO^10, 11^. Although studies using optical coherence tomography angiography (OCTA) suggested potential predictors of anti-VEGF requirement over 1 year^12–15^, retinal hemorrhage and severe ME often cause difficulties in evaluating images at the initial visit. Sectional optical coherence tomography (OCT) images at the initial visit may allow for visual acuity (VA) prediction^16–19^; however, prediction of ME recurrence is difficult at the initial visit^20^.

We reviewed the OCT images of eyes with BRVO and ME in our clinic and found that the eyes with a smooth border between the inner nuclear layer (INL) and the outer plexiform layer (OPL) seemed to have fewer ME recurrences, and the eyes with a non-smooth border (jagged border) received more anti-VEGF injections. Hence, this study aimed to determine whether the status of the border between the INL and OPL at the initial visit correlates with the total number of recurrences of ME secondary to BRVO and with VA at the 12-month visit.

## Results

### Patient characteristics

Of the 65 eyes of 65 patients with ME secondary to treatment-naïve BRVO that were included initially, 28 eyes did not meet the study criteria: over 6 months from BRVO onset (n = 3); hemicentral retinal vein occlusion (RVO) (n = 1); epiretinal membrane (n = 3); low OCT image quality due to dense hemorrhages (n = 3); progressive cataract at the initial visit (n = 3); additional treatments during the study period, such as cataract surgery (n = 3), laser photocoagulation (n = 3), and treatment with sub-Tenon triamcinolone acetonide injection (n = 4); and follow-up periods less than 12 months (n = 5). Thus, 37 eyes of 37 patients (mean age, 71.5 ± 8.1 years; range 55–88) met the study criteria for analysis.

Based on the OCT images at the initial visit, all 37 eyes (100%) had cystoid spaces in the OPL; 30 eyes (81.1%) had cystoid spaces in both the INL and OPL, and seven eyes (18.9%) in the OPL only. None of the patients had cystoid spaces in the INL only. The intraclass correlation coefficients (ICCs) of the two examiners (H. S. and R. K.) for jagged ratio (JR), INL area, and outer retina area were high (0.95, 0.92, and 0.97, respectively). Three examiners (H. S., R. K., and M. K.) independently measured the number of cystoid spaces in the INL, and the ICC among the three examiners was 0.8998. Table 1 shows that patient characteristics.

**Table 1:** Patient characteristics and optical coherence tomography findings at the initial visit.

**Baseline characteristics**	
No. eyes	37
Age (years)	71.5 ± 8.1
Sex (male/female)	11/26
Eye (right/left)	17/20
Hypertension, no. (%)	26 (70.3)
Diabetes mellitus, no. (%)	2 (5.4)
Duration of symptoms before initial treatment (weeks)	7.8 ± 7.0
LogMAR visual acuity	0.53 ± 0.4
Central subfield thickness (μm)	508.4 ± 154.8
Location of intraretinal cystoid space (INL/OPL/both), no.	0/7/30
Subretinal fluid, no. (%)	14 (37.8)
Subtype (macular/major), no.	10/27
Perfusion status (perfused/ischemic), no.	15/21
Jagged ratio	1.1 ± 0.07
No. INL cystoid spaces	3.8 ± 3.1
INL area (mm^2^)	0.29 ± 0.09
Outer retina area (mm^2^)	1.21 ± 0.37
Drugs injected (Ranibizumab/Aflibercept), no.	18/19

Fluorescein angiography was not performed in 1 eye. The data are expressed as the mean ± standard deviation.

*INL* inner nuclear layer, *logMAR* logarithm of the minimum angle of resolution, *No.* number, *OPL* outer plexiform layer.

### Prediction of the total number of anti-VEGF injections over 1 year

The total number of anti-VEGF injections over the 1-year follow-up period was 4.1 ± 2.1 (range 1–9). Eighteen eyes were treated with ranibizumab and 19 with aflibercept. No drug switching was observed during the study period. No differences in the total number of anti-VEGF injections between the eyes treated with ranibizumab and those treated with aflibercept were found (4.0 ± 0.5 vs. 4.1 ± 0.5, *P* = 0.88).

**Figure 1:**
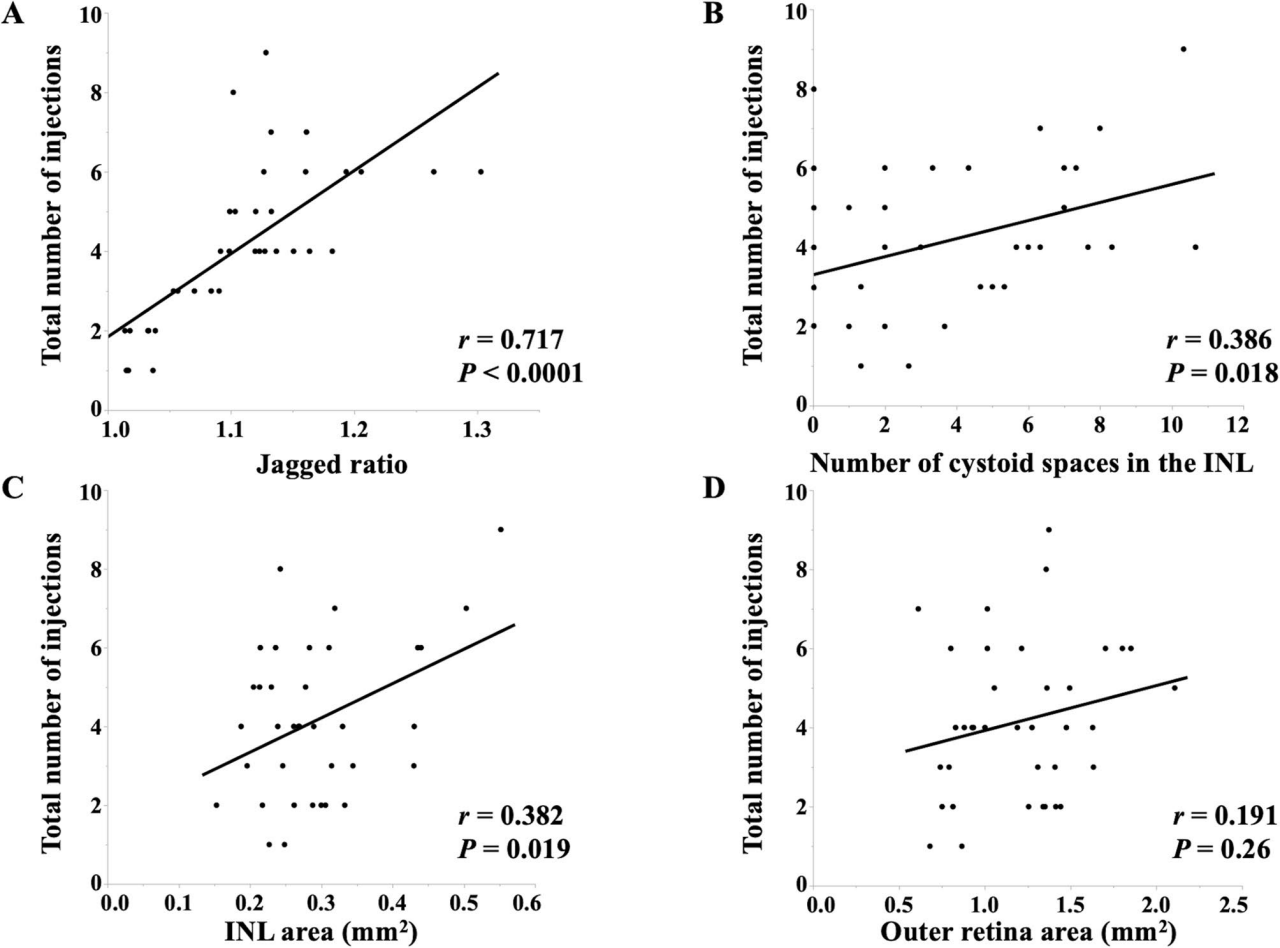
The scatterplots show the correlations between the parameters at the initial visit and the total number of anti-vascular endothelial growth factor (VEGF) injections over 1-year follow-up period. The jagged ratio (*r* = 0.717, *P* < 0.0001), number of cystoid spaces in the inner nuclear layer (INL) (*r* = 0.386, *P* = 0.018), and INL area (*r* = 0.382, *P* = 0.019) were significantly correlated with the total number of anti-VEGF injections. Figure is created using JMP version 15.2.1 (SAS Institute, Inc., Cary, NC, USA, available at https://www.jmp.com/ja_jp/home.html).

Univariate linear regression analysis for predicting the total number of anti-VEGF injections demonstrated that the JR (*r* = 0.717, *P* < 0.0001), the number of cystoid spaces in the INL (*r* = 0.386, *P* = 0.018), and the INL area (*r* = 0.382, *P* = 0.019) are significantly correlated with the total number of anti-VEGF injections (Fig. 1). Duration of symptoms before initial treatment, number of cystoid spaces in the INL, and INL area were correlated with both JR and the number of anti-VEGF injections (all *P* < 0.1) and, thus, were identified as potential confounders (Supplementary Tables S1, S2). In the multivariate linear regression analysis, including the JR and the three potential confounders, the JR was significantly correlated with the total number of anti-VEGF injections (*P* < 0.0001; standardized coefficient, 0.713) (Table 2).

**Table 2:** Factors affecting the total number of anti-vascular endothelial growth factor injections over 1-year follow-up period in multivariate linear regression analysis.

Variables at the initial visit	Coefficient (95% CI)	*P* value	Standardized coefficient
Jagged ratio	21.7 (12.8 to 30.5)	< 0.0001	0.713
Duration of symptoms before initial treatment (weeks)	− 0.025 (− 0.11 to 0.061)	0.55	− 0.085
No. INL cystoid spaces	− 0.047 (− 0.29 to 0.2)	0.7	− 0.07
INL area (mm^2^)	5.0 (− 3.0 to 13.0)	0.21	0.214

*INL* inner nuclear layer, *CI* confidence interval, *No.* number.

Moreover, univariate regression analysis demonstrated that the drug type is significantly correlated with the JR (*P* = 0.037) (Supplementary Table S2) but not with the total number of anti-VEGF injections (*P* = 0.88) (Supplementary Table S1). Then we further performed a multivariate regression analysis including the two variables (JR and drug type) for predicting the total number of anti-VEGF injections to adjust the influence of drug difference. The result showed that JR is still a significant predictor of the total number of anti-VEGF injections (*P* < 0.0001; standardized coefficient, 0.803).

Of the 37 eyes, nine eyes (24.3%) receiving two or fewer injections over the 1-year follow-up tended to have a smooth border and thus a lower JR. In the subgroup analysis, the mean JR was significantly lower in the nine eyes receiving two or fewer injections than in the 28 eyes receiving three or more injections (1.02 ± 0.01 vs. 1.13 ± 0.06, *P* < 0.0001).

### Correlation between JR and OCT parameters at the initial visit

**Figure 2:**
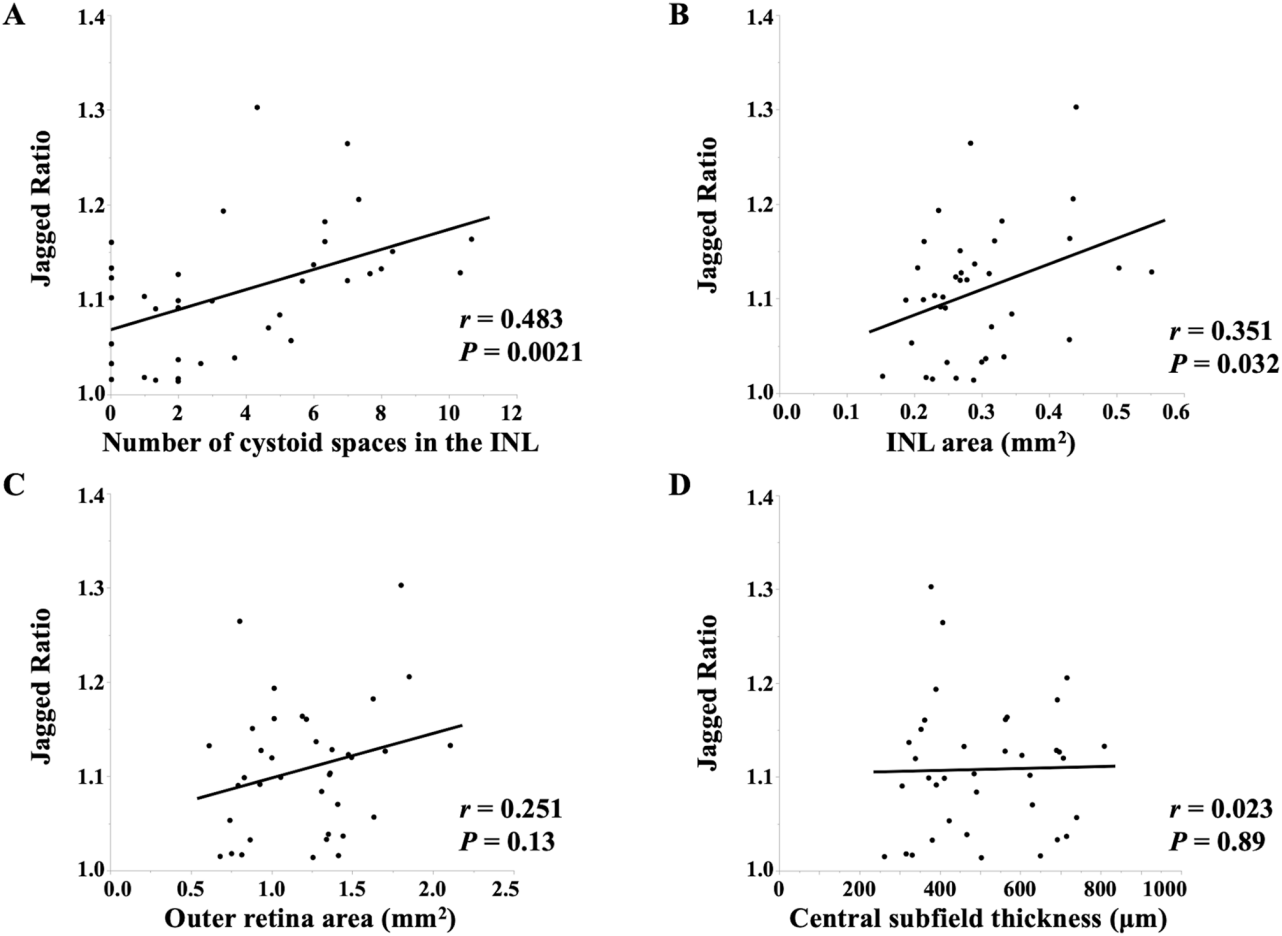
The scatterplots show the correlations between jagged ratio (JR) and optical coherence tomography parameters at the initial visit. The number of cystoid spaces in the inner nuclear layer (INL) (*r* = 0.483, *P* = 0.0021) and INL area (*r* = 0.351, *P* = 0.032) were significantly correlated with JR. Figure is created using JMP version 15.2.1 (SAS Institute, Inc., Cary, NC, USA, available at https://www.jmp.com/ja_jp/home.html).

We evaluated the relationship between the JR and OCT parameters to explore the mechanism of the jagged border formation. The number of cystoid spaces in the INL (*r* = 0.483, *P* = 0.0021) and the INL area (*r* = 0.351, *P* = 0.032) were significantly correlated with the JR, whereas the outer retina area and the central subfield thickness (CST) was not (Fig. 2).

Jagged borders were also observed in five of seven eyes without cystoid spaces in the INL at the initial visit. In these seven eyes, we further analyzed the relationship between JR and OCT parameters at the time of the first ME recurrence. The patients who received a small number of injections tended to have a lower JR at the initial visit, whereas those who needed frequent injections tended to have a higher JR (Table 3). Duration of symptoms before initial treatment was 8.4 ± 9.0 weeks in five eyes with a JR > 1.05.

**Table 3:** Characteristics of seven cases with cystoid spaces only in the outer plexiform layer at the initial visit.

Case	Jagged ratio	Duration before initial recurrence (weeks)	No. cystoid spaces in INL at initial recurrence	Total number of anti-VEGF injections
1	1.033048	16	0	2
2	1.015892	8	0	2
3	1.053238	12	4	3
4	1.150741	8	7	6
5	1.101775	4	6	8
6	1.132699	16	5	5
7	1.123045	8	6	4
Mean ± SD	1.09 ± 0.05	10.3 ± 4.5	4.0 ± 2.9	4.3 ± 2.2

*SD* standard deviation, *INL* inner nuclear layer, *VEGF* vascular endothelial growth factor, *No.* number.

### JR between intraretinal cystoid space locations

The mean JR in eyes with cystoid spaces in both the INL and OPL was 1.11 ± 0.07 and that in eyes with cystoid spaces in the OPL only was 1.09 ± 0.05. No significant difference in the JR between locations of the cystoid spaces was found (*P* = 0.41).

### VA prediction at the 12-month visit

Univariate linear regression analysis for predicting postoperative VA showed no correlation between the JR and postoperative VA at the 12-month visit (*P* = 0.11). Age, duration of symptoms before initial treatment, logarithm of the minimum angle of resolution (logMAR) VA, CST, outer retina area, and drug type were significant variables (all *P* < 0.05) (Supplementary Table S3).

### Representative cases

**Figure 3:**
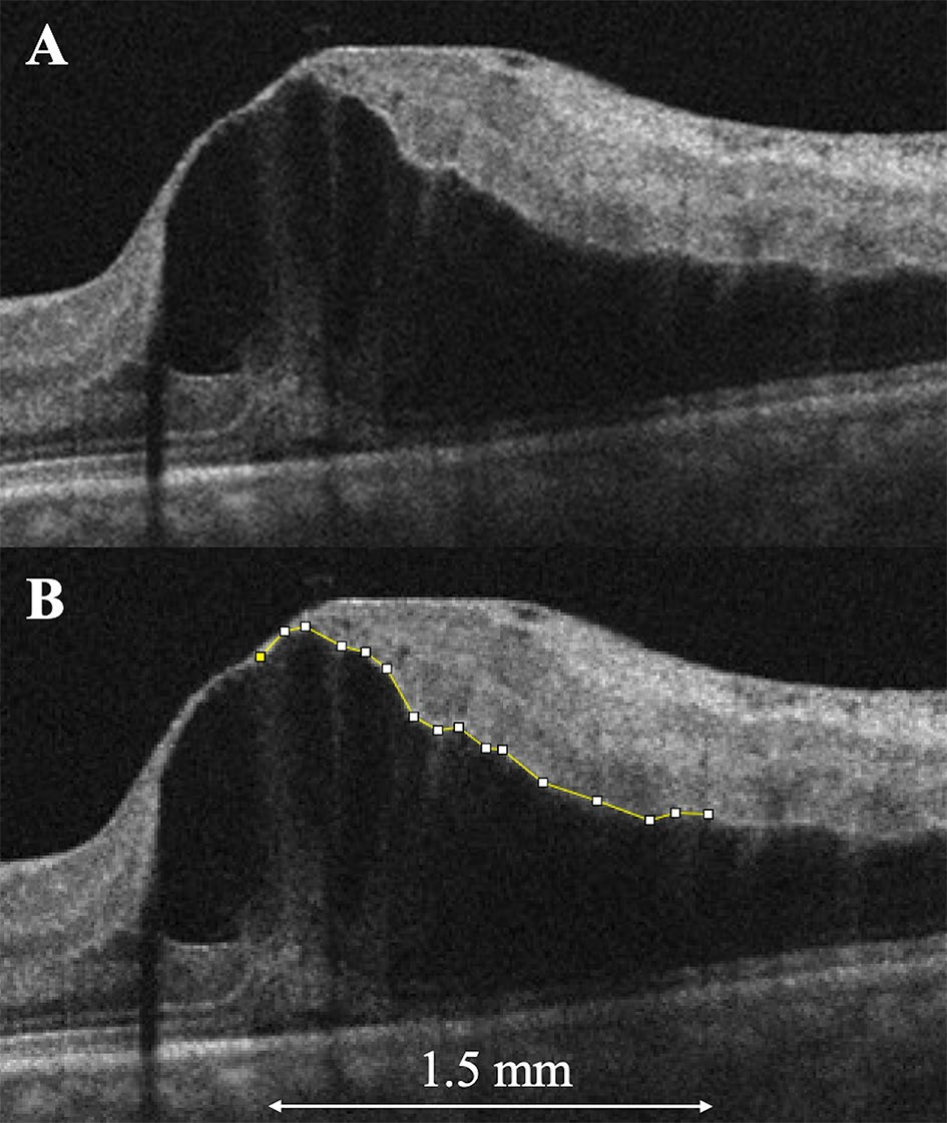
A spectral-domain optical coherence tomography (OCT) image of an eye with branch retinal vein occlusion of a 77-year-old woman who required only one anti-vascular endothelial growth factor injection over 1-year follow-up period. (**A**) An OCT image that ran vertically through the fovea at the initial visit shows macular edema, with large cystoid spaces in the outer plexiform layer (OPL). (**B**) The border between the inner nuclear layer and OPL is relatively smooth (segmented line). In this case, the jagged ratio is 1.01. Figure is created using ImageJ software version 1.53a (National Institutes of Health, Bethesda, Maryland, USA, available at https://imagej.nih.gov/ij/).

Case 1 was a 77-year-old woman who presented with visual loss due to ME secondary to BRVO in the left eye. At the initial visit, ME was observed on OCT images, which showed large cystoid spaces in the OPL and a small amount of subretinal fluid (Fig. 3A). The border between the INL and OPL was relatively smooth (Fig. 3B), and the JR was 1.01. After one anti-VEGF injection at the initial visit, ME was not observed until the end of the 12-month study period.

**Figure 4:**
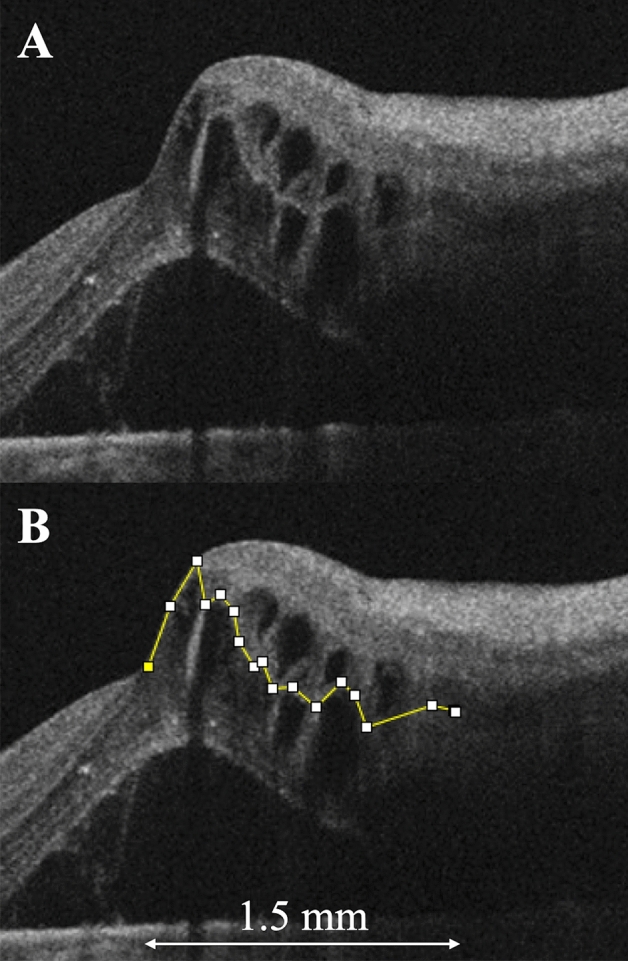
An optical coherence tomography (OCT) image of an eye with branch retinal vein occlusion of a 64-year-old woman who required six anti-vascular endothelial growth factor injections over 1-year follow-up period. (**A**) An OCT image that ran vertically through the fovea at the initial visit shows macular edema, with relatively large cystoid spaces in the inner nuclear layer (INL), large cystoid spaces in the outer plexiform layer (OPL), and subretinal detachment. (**B**) The border between the INL and OPL is not smooth (segmented line). In this case, the jagged ratio is 1.21. Figure is created using ImageJ software version 1.53a (National Institutes of Health, Bethesda, Maryland, USA, available at https://imagej.nih.gov/ij/).

Case 2 was a 64-year-old woman who presented with visual loss due to ME secondary to BRVO in the left eye. At the initial visit, ME was noted on OCT images, which showed relatively large cystoid spaces in the INL, large cystoid spaces in the OPL, and subretinal detachment (Fig. 4A). The border between the INL and OPL was not smooth (Fig. 4B), and the JR was 1.21. The patient was treated with six anti-VEGF injections over the 12-month study period.

## Discussion

In this study, we quantitatively assessed the smoothness of the border between the INL and OPL and examined JR as a new and potential predictor of ME recurrence. Over the 1-year follow-up period, we found that the eyes with a higher JR had a greater number of anti-VEGF injections, and the eyes receiving two or fewer injections had a lower JR, thereby suggesting that JR may be a novel predictor of fewer ME recurrences during anti-VEGF treatment of BRVO.

Formation of a jagged border (high JR) was significantly associated with a greater number of cystoid spaces in the INL and a larger INL area, which in turn suggests that INL swelling results in a jagged border between the INL and OPL. Nevertheless, eyes without cystoid spaces in the INL at the initial visit sometimes have jagged borders. When we assessed the relationship between JR and the number of cystoid spaces in the INL at the initial ME recurrence, eyes with a higher JR tended to have a greater number of cystoid spaces in the INL (Table 3). In summary, a jagged border was observed not only in eyes with more cystoid spaces in the INL and a larger area of the INL but also in some eyes with no cystoid spaces in the INL at the initial visit but INL cysts that developed later, suggesting that estimating JR predicts ME recurrences at an earlier stage than assessing INL cysts.

We speculated that the jagged border formation is associated with retinal deep capillaries. Recent studies have reported on the mechanisms of persistent ME and have investigated potential predictors of frequent anti-VEGF injection in eyes with BRVO^10–15, 21^. Deep capillary loss detected by OCTA may be associated with persistent ME^12, 13^. Supposing that deep capillaries drain interstitial fluid from the retina, ME more likely occurs in eyes without deep capillaries^21^. Moreover, capillary dilation and collateral vessel formation, which primarily occur in deep capillary plexus, are also observed in eyes with recurrent ME^14, 15^. Our study did not assess these angiographic factors; nonetheless, we speculate that deep capillaries, which run between the INL and OPL^22^, possibly contribute to jagged border formation. Further investigations of the relationship between JR and angiographic parameters are needed.

Recurrent ME may lead to worse VA^23^; thus, we also investigated the relationship between JR and VA at 12 months postoperatively. We found no correlation between JR and 12-month VA. The patients in this study were strictly treated with PRN anti-VEGF injection with a monthly visit to the hospital and observation period was only 1 year, which could explain the minimal effect of the number of ME recurrences on postoperative VA at 12 months in our study.

This study has several limitations. First, we included eyes treated with two different drugs: 18 eyes were treated with ranibizumab; 19 eyes, with aflibercept. Although the drug type was not associated with the total number of anti-VEGF injections, the effect of drug difference should be of concern^24, 25^. Second, sampling bias inherent to the retrospective study design and small sample size exists. Lastly, we used a single OCT B-scan image of a vertical section through the fovea to measure the OCT parameters and had all the parameters manually measured by the examiners. Although the ICCs of all the parameters were sufficient, more robust measurements, such as automated segmentation tools or three-dimensional detection of cystoid spaces, are desirable.

Despite the limitations, our study has strengths. The jagged border could be assessed using a single vertical OCT B-scan image, which could be obtained by an OCT device in a clinical setting. In addition, the border between the INL and OPL was almost always observed at the initial visit. All these help predict recurrent ME and may facilitate the selection of treatment options, such as PRN anti-VEGF injection, treat-and-extend regimen, and loading dose administration. Although the jagged border mechanism requires further investigation in future studies, we believe that a smooth border is a new biomarker that may be useful in the treatment of patients with BRVO in routine clinical practice.

## Methods

### Patients and examinations

This retrospective observational study included consecutive patients who visited the Department of Ophthalmology, Aichi Medical University Hospital, from May 25, 2016, to October 1, 2018, and had visual impairment associated with treatment-naïve BRVO. The institutional review board of Aichi Medical University approved the study protocol (reference number: 2019-082), which adhered to the tenets of the Declaration of Helsinki. Informed consent was obtained from all participants included in this study. We reviewed the medical and ocular histories of the patients in detail.

The inclusion criteria included patients with ME within 6 months after BRVO onset and who were treated with anti-VEGF drugs (either 0.5 mg/0.05 mL ranibizumab or 2.0 mg/ 0.05 mL aflibercept [Eylea, Regeneron Pharmaceuticals, Inc., Tarrytown, NY]) in one + PRN regimen over 12 months with a monthly visit to the hospital. According to our institutional protocol for the treatment of ME secondary to BRVO, anti-VEGF drugs were strictly administered when the CST exceeded 300 μm. The exclusion criteria were the presence of hemicentral RVO, epiretinal membranes, macular holes, diabetic retinopathy, age-related macular degeneration, and poor OCT images because of significant cataract or retinal hemorrhage. Patients who had an active ocular infection or intraocular inflammation; had undergone surgeries during the current study such as cataract surgery, vitrectomy, and laser photocoagulation; or were treated with a sub-Tenon’s capsule injection of triamcinolone acetonide were also excluded.

During the study period, all patients underwent ophthalmic examinations, including best-corrected visual acuity (BCVA) measurement using a Landolt C chart and evaluations by indirect ophthalmoscopy, ultra-widefield fundus camera (California, Optos PLC, Dunfermline, UK), and OCT (Cirrus-HD 5000, Carl Zeiss Meditec, Dublin, CA). All but one patient underwent FA examination using an ultra-widefield fundus camera. Nonperfusion area (NPA) was measured on FA images, which were obtained 30 to 40 s after dye administration, and eyes with an NPA smaller than five disc diameters were considered to be perfused^1^. BRVO subtype (i.e., major or macular) was determined using an ultra-widefield fundus camera and based on the vein occluded: major BRVO is due to occlusion of one of the four major branch retinal veins; macular BRVO, occlusion of the veins from the macular region^26^.

### Assessment of the border and JR

**Figure 5:**
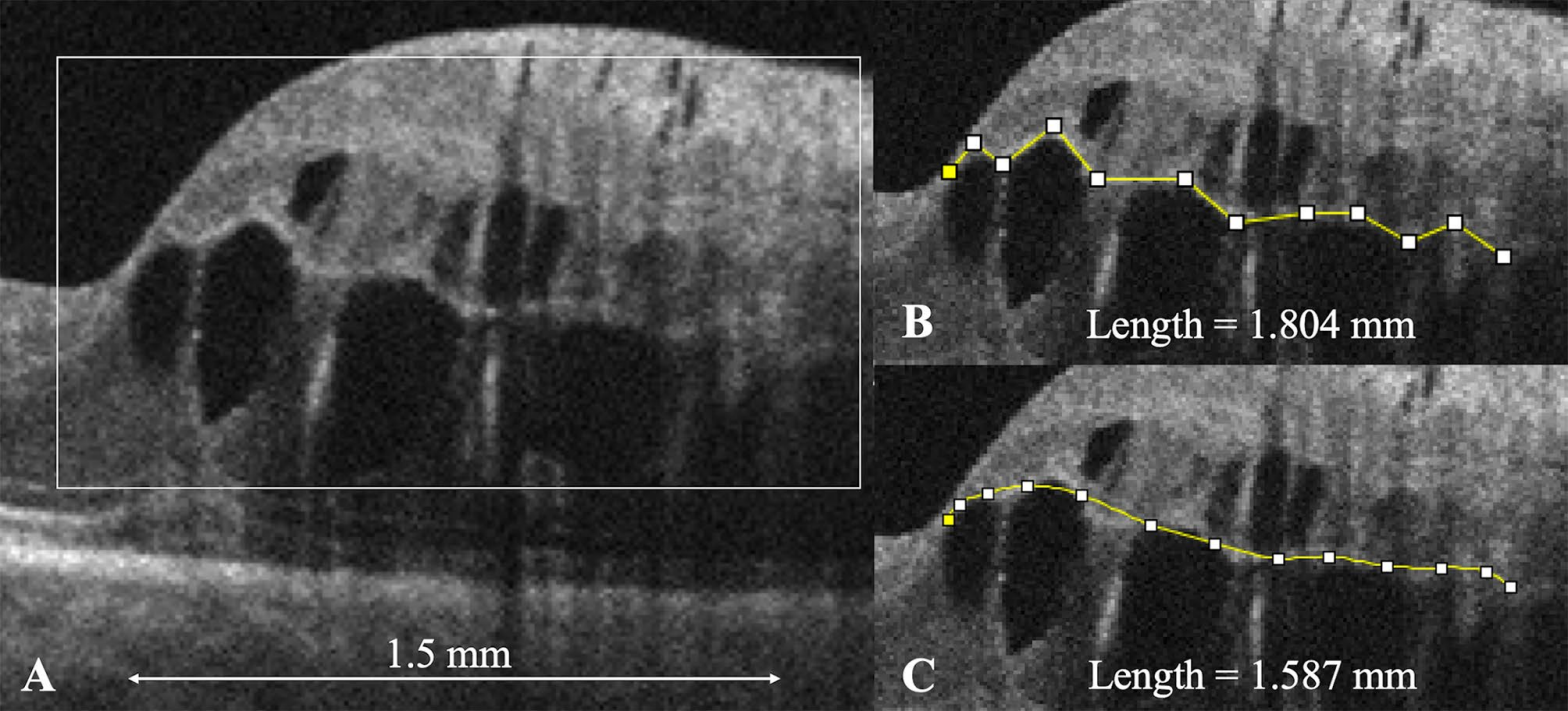
The jaggedness of the border between the inner nuclear layer (INL) and the outer plexiform layer (OPL) in eyes with macular edema (ME) secondary to branch retinal vein occlusion. (**A**) At the initial visit, an optical coherence tomography (OCT) vertical scan through the fovea shows ME. (**B,C**) Magnified images of the box in (**A**). (**B**) We manually traced the border (segmented line) between the INL and OPL on the images within 1.5 mm from the fovea using ImageJ software. The border length in this case was 1.804 mm. (**C**) We also manually drew a spline curve through the middle points of the border using the “Spline Fit” mode in the software and measured its length (1.587 mm). The jagged ratio (JR) then was calculated as the border length/spline curve length; the JR is 1.14 in this case. Figure is created using ImageJ software version 1.53a (National Institutes of Health, Bethesda, Maryland, USA, available at https://imagej.nih.gov/ij/).

To evaluate the smoothness of the border between the INL and the OPL, we obtained an OCT B-scan image vertically across the fovea at the initial visit and measured the border length on the affected side at 1.5 mm from the fovea using ImageJ software version 1.53a (National Institutes of Health, Bethesda, MD, available at http://imagej.nih.gov/ij)^27^ (Fig. 5). First, we dragged and dropped an OCT image onto the application icon on a computer. Second, using the “Set Scale” mode of the application, we measured the distance between the ends of the images and identified 1.5 mm as the actual distance in pixels (Fig. 5A). Third, using the “Segmented Line” mode of the application, we manually traced the border on the images and measured the border length (Fig. 5B).

To evaluate the extent of the jaggedness of the border, we drew a smooth line as a reference by drawing a spline curve through the middle points of each side on the polygonal line using the “Spline Fit” mode of the application (Fig. 5C) and measured the spline curve length. Finally, we defined JR as the extent of border jaggedness using the following formula: JR = border length/spline curve length. Using the number of anti-VEGF injections as the number of ME recurrences, we evaluated the correlation between the JR at the initial visit and the number of anti-VEGF injections in 1 year. Two examiners (H. S. and R. K.) independently measured the border and spline curve lengths and calculated the JRs; the mean JR of the two examiners was analyzed.

### Factors affecting ME recurrences

**Figure 6:**
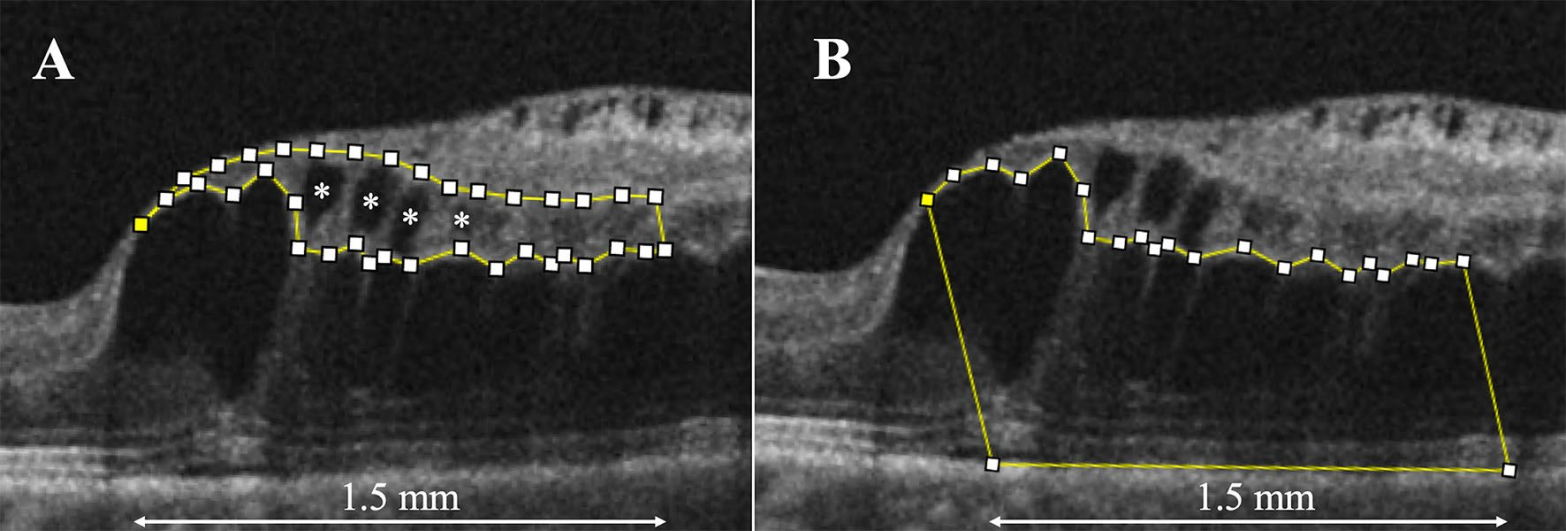
Assessment of the inner nuclear layer (INL) area, the number of INL cystoid spaces, and the outer retina area associated with branch retinal vein occlusion. (**A**) The INL area (between the inner and outer borders of the INL) was manually outlined within the affected 1.5-mm vertical optical coherence tomography (OCT) line from the fovea. The INL area is 0.263 mm^2^ in this case. The number of INL cystoid spaces (asterisks) also was counted on this image. (**B**) The outer retina area (between the inner aspect of the outer plexiform layer and the inner border of the retinal pigment epithelium) was manually outlined and is 1.027 mm^2^ in this case. These OCT parameters were measured within the affected 1.5-mm vertical line from the fovea (double-headed arrow). Figure is created using ImageJ software version 1.53a (National Institutes of Health, Bethesda, Maryland, USA, available at https://imagej.nih.gov/ij/).

To evaluate other factors affecting the total number of anti-VEGF injections over 1 year, we examined other OCT parameters, including the INL area (Fig. 6A), the number of cystoid spaces in the INL (Fig. 6A, asterisks), and the outer retina area (Fig. 6B) at the initial visit. The area between the inner and outer borders of the INL was outlined manually on a vertical OCT scan of the affected side using the “Polygon Selection” mode in the ImageJ software (Fig. 6A). The number of cystoid spaces in the INL within 1.5 mm from the fovea on the affected side was counted in the OCT vertical scan (Fig. 6A). The cystoid spaces were also observed in the OPL; however, those at the borders were extremely large to be counted. Similarly, the outer retina area was outlined manually between the inner aspect of the OPL and the inner border of the retinal pigment epithelium (Fig. 6B). The outer retina area was also measured within the 1.5-mm vertical line from the fovea using ImageJ software. Two examiners (H. S. and R. K.) independently measured the INL and outer retina areas, and the mean values of the two examiners were analyzed. Three examiners (H. S., R. K., and M. K.) independently counted the number of cystoid spaces in the INL, and the mean values of the three examiners were analyzed.

### Statistical analysis

A biostatistician (K. M.) performed the statistical analyses using SAS version 9.4 software (; SAS Institute, Inc., Cary, NC, USA). All values are expressed as the mean ± standard deviation. Decimal BCVA was converted to the logMAR units for analysis. The ICCs of the two examiners were calculated to assess the intergrader agreement on the JR, INL area, and outer retina area in the OCT images, and the ICCs of the three examiners were calculated to assess the intergrader agreement on the number of cystoid spaces in the INL.

Considering the need for anti-VEGF injections in recurrent ME, we evaluated the correlation between the JR in the OCT images at the initial visit and the number of anti-VEGF injections as well as the factors affecting ME recurrences. Pearson correlation coefficient was used in the univariate analysis of the relationships between potential predictors and outcomes as well as among potential predictors. Variables with a *P* value of 0.1 or less in both the univariate analysis of the JR and that of the outcomes were considered potential confounders^28^. Subsequently, we performed a multivariate linear regression analysis including not only JR but also all potential confounders to explore the predictive value of the JR. *P* < 0.05 was considered as statistically significant.

## Supplementary Information

Supplementary Information.

## References

[1] The Branch Vein Occlusion Study Group (1984). Argon laser photocoagulation for macular edema in branch vein occlusion. Am. J. Ophthalmol..

[2] Glacet-Bernard A, Coscas G, Chabanel A (1996). Prognostic factors for retinal vein occlusion: Prospective study of 175 cases. Ophthalmology.

[3] Rogers SL, McIntosh RL, Lim L (2010). Natural history of branch retinal vein occlusion: An evidence-based systematic review. Ophthalmology.

[4] Campochiaro PA, Heier JS, Feiner L (2010). Ranibizumab for macular edema following branch retinal vein occlusion: Six-month primary end point results of a phase III study. Ophthalmology.

[5] Heier JS, Campochiaro PA, Yau L (2012). Ranibizumab for macular edema due to retinal vein occlusions: Long-term follow-up in the HORIZON trial. Ophthalmology.

[6] Campochiaro PA, Sophie R, Pearlman J (2014). Long-term outcomes in patients with retinal vein occlusion treated with ranibizumab: The RETAIN study. Ophthalmology.

[7] Campochiaro PA, Clark WL, Boyer DS (2015). Intravitreal aflibercept for macular edema following branch retinal vein occlusion: The 24-week results of the VIBRANT study. Ophthalmology.

[8] Miwa Y, Muraoka Y, Osaka R (2017). Ranibizumab for macular edema after branch retinal vein occlusion: One initial injection versus three monthly injections. Retina.

[9] Murata T, Kondo M, Inoue M (2021). The randomized ZIPANGU trial of ranibizumab and adjunct laser for macular edema following branch retinal vein occlusion in treatment-naïve patients. Sci. Rep..

[10] Sakimoto S, Kamei M, Suzuki M (2013). Relationship between grades of macular perfusion and foveal thickness in branch retinal vein occlusion. Clin. Ophthalmol..

[11] Yoo JH, Ahn J, Oh J (2017). Risk factors of recurrence of macular oedema associated with branch retinal vein occlusion after intravitreal bevacizumab injection. Br. J. Ophthalmol..

[12] Tsuboi K, Ishida Y, Kamei M (2017). Gap in capillary perfusion on optical coherence tomography angiography associated with persistent macular edema in branch retinal vein occlusion. Investig. Ophthalmol. Vis. Sci..

[13] Hasegawa T, Murakawa S, Maruko I (2019). Correlation between reduction in macular vessel density and frequency of intravitreal ranibizumab for macular oedema in eyes with branch retinal vein occlusion. Br. J. Ophthalmol..

[14] Tsuboi K, Sasajima H, Kamei M (2019). Collateral vessels in branch retinal vein occlusion: Anatomic and functional analyses by OCT angiography. Ophthalmol. Retina.

[15] Kogo T, Muraoka Y, Uji A (2021). Angiographic risk factors for recurrence of macular edema associated with branch retinal vein occlusion. Retina.

[16] Kondo M, Kondo N, Ito Y (2009). Intravitreal injection of bevacizumab for macular edema secondary to branch retinal vein occlusion: Results after 12 months and multiple regression analysis. Retina.

[17] Jaissle GB, Szurman P, Feltgen N (2011). Predictive factors for functional improvement after intravitreal bevacizumab therapy for macular edema due to branch retinal vein occlusion. Graefes Arch. Clin. Exp. Ophthalmol..

[18] Hasegawa T, Ueda T, Okamoto M, Ogata N (2014). Presence of foveal bulge in optical coherence tomographic images in eyes with macular edema associated with branch retinal vein occlusion. Am. J. Ophthalmol..

[19] Mimouni M, Segev O, Dori D (2017). Disorganization of the retinal inner layers as a predictor of visual acuity in eyes with macular edema secondary to vein occlusion. Am. J. Ophthalmol..

[20] Suzuki M, Nagai N, Minami S (2020). Predicting recurrences of macular edema due to branch retinal vein occlusion during anti-vascular endothelial growth factor therapy. Graefes Arch. Clin. Exp. Ophthalmol..

[21] Spaide RF (2016). Retinal vascular cystoid macular edema: Review and new theory. Retina.

[22] Nesper PL, Fawzi AA (2018). Human parafoveal capillary vascular anatomy and connectivity revealed by optical coherence tomography angiography. Investig. Ophthalmol. Vis. Sci..

[23] Iftikhar M, Mir TA, Hafiz G (2019). Loss of peak vision in retinal vein occlusion patients treated for macular edema. Am. J. Ophthalmol..

[24] Hykin P, Prevost AT, Vasconcelos JC (2019). Clinical effectiveness of intravitreal therapy with ranibizumab vs aflibercept vs bevacizumab for macular edema secondary to central retinal vein occlusion: A randomized clinical trial. JAMA Ophthalmol..

[25] Casselholm de Salles M, Amrén U, Kvanta A, Epstein DL (2019). Injection frequency of aflibercept versus ranibizumab in a treat-and-extend regimen for central retinal vein occlusion: A randomized clinical trial. Retina.

[26] Hayreh SS, Zimmerman MB (2015). Fundus changes in branch retinal vein occlusion. Retina.

[27] Schneider CA, Rasband WS, Eliceiri KW (2012). NIH image to ImageJ: 25 years of image analysis. Nat. Methods..

[28] Austin PC, Steyerberg EW (2015). The number of subjects per variable required in linear regression analyses. J. Clin. Epidemiol..

