# Supplementary material for: Smooth borders between inner nuclear layer and outer plexiform layer predict fewer macular edema recurrences in branch retinal vein occlusion
Source: Sci Rep. 2021 Aug 6;11:15987. doi: 10.1038/s41598-021-95501-w (PMC8346557; doi:10.1038/s41598-021-95501-w)
Supplement: Supplementary file 1 — Supplementary Information. [file 41598_2021_95501_MOESM1_ESM.docx]

**Smooth Borders Between Inner Nuclear Layer and Outer Plexiform Layer Predict Fewer Macular Edema Recurrences in Branch Retinal Vein Occlusion**

*Hirofumi Sasajima^1^****^*^,*** *Kotaro Tsuboi^2,3^, Rokuki Kiyosawa^2^, Akira Fukutomi^2^, Kenta Murotani^4^ and Motohiro Kamei^2^*

^1^Department of Ophthalmology, Shinseikai Toyama Hospital, Imizu, Toyama, Japan

^2^Department of Ophthalmology, Aichi Medical University, Nagakute, Aichi, Japan

^3^Casey Eye Institute, Oregon Health & Science University, Portland, United States

^4^Biostatistics Center, Graduate School of Medicine, Kurume University, Fukuoka, Japan

Corresponding author; email: [hiro.sasa1228@icloud.com](mailto:hiro.sasa1228@icloud.com)

| Parameters at initial visit | Coefficient (95% CI) | *P* Value |
| --- | --- | --- |
| Age (years) | 0.007 (-0.08 to 0.09) | 0.87 |
| Sex (female) | -0.014 (-1.54 to 1.5) | 0.99 |
| Eye (left) | -0.068 (-1.47 to 1.3) | 0.92 |
| Hypertension (yes) | -1.18 (-2.65 to 0.29) | 0.11 |
| Diabetes mellitus (yes) | 1.5 (-1.54 to 4.54) | 0.32 |
| Duration of symptoms before initial treatment (weeks) | 0.084 (-0.013 to 0.18) | 0.087 |
|  |  |  |
| LogMAR visual acuity | -0.53 (-2.28 to 1.23) | 0.55 |
| Central subfield thickness (μm) | 0.0028 (-0.0017 to 0.0072) | 0.22 |
| Subretinal fluid (present) | 0.44 (-0.99 to 1.87) | 0.53 |
| Subtype (major) | 1.48 (-0.004 to 2.97) | 0.051 |
| Perfusion status (perfused) | -1.2 (-2.55 to 0.15) | 0.079 |
| Jagged ratio | 21.8 (14.5 to 29.0) | < 0.0001 |
| No. INL cystoid spaces | 0.26 (0.047 to 0.47) | 0.018 |
| INL area (mm^2^) | 8.91 (1.51 to 16.3) | 0.019 |
| Outer retina area (mm^2^) | 1.1 (-0.83 to 3.0) | 0.26 |
| Drugs injected (aflibercept) | 0.11 (-1.31 to 1.5) | 0.88 |

**Supplementary Table S1.** Relationships between the total number of anti-vascular endothelial growth factor injections over 1 year and the variables obtained at the initial visit. INL, inner nuclear layer; logMAR, logarithm of the minimum angle of resolution; CI, confidence interval; No. = number.

| Parameters at initial visit | Coefficient (95% CI) | *P* Value |
| --- | --- | --- |
| Age (years) | -0.00061 (-0.0035 to 0.0023) | 0.67 |
| Sex (female) | -0.014 (-0.065 to 0.037) | 0.59 |
| Eye (left) | -0.0053 (-0.052 to 0.042) | 0.82 |
| Hypertension (yes) | -0.027 (-0.077 to 0.023) | 0.29 |
| Diabetes mellitus (yes) | 0.019 (-0.084 to 0.12) | 0.71 |
| Duration of symptoms before initial treatment (weeks) | 0.0044 (0.0014 to 0.0074) | 0.0057 |
| LogMAR visual acuity | -0.031 (-0.089 to 0.024) | 0.26 |
| Central subfield thickness (μm) | 0.00001 (-0.0001 to 0.00016) | 0.89 |
| Subretinal fluid (present) | -0.0045 (-0.053 to 0.044) | 0.85 |
| Subtype (major) | 0.034 (-0.017 to 0.085) | 0.18 |
| Perfusion status (perfused) | -0.033 (-0.08 to 0.013) | 0.15 |
| No. INL cystoid spaces | 0.0011 (0.0041 to 0.017) | 0.0021 |
| INL area (mm^2^) | 0.27 (0.023 to 0.52) | 0.032 |
| Outer retina area (mm^2^) | 0.047 (-0.015 to 0.11) | 0.13 |
| Drugs injected (aflibercept) | 0.047 (0.003 to 0.091) | 0.037 |

**Supplementary Table S2.** Relationships between jagged ratio and variables obtained at the initial visit. INL, inner nuclear layer; logMAR, logarithm of the minimum angle of resolution; CI, confidence interval; No. = number.

| Parameters at initial visit | Coefficient (95% CI) | *P* Value |
| --- | --- | --- |
| Age (years) | 0.016 (0.006 to 0.026) | 0.0027 |
| Sex (female) | -0.013 (-0.21 to 0.19) | 0.9 |
| Eye (left) | -0.094 (-0.27 to 0.084) | 0.29 |
| Hypertension (yes) | -0.1 (-0.3 to 0.093) | 0.29 |
| Diabetes mellitus (yes) | -0.19 (-0.58 to 0.21) | 0.34 |
| Duration of symptoms before initial treatment (weeks) | 0.011 (-0.0018 to 0.023) | 0.049 |
| LogMAR visual acuity | 0.39 (0.2 to 0.57) | 0.0001 |
| Central subfield thickness (μm) | 0.00073 (0.0002 to 0.0013) | 0.0088 |
| Subretinal fluid (present) | 0.14 (-0.037 to 0.32) | 0.11 |
| Subtype (major) | 0.12 (-0.082 to 0.32) | 0.24 |
| Perfusion status (perfused) | -0.16 (-0.34 to 0.013) | 0.068 |
| Jagged ratio | 1.0 (-0.24 to 2.3) | 0.11 |
| No. INL cystoid spaces | 0.0068 (-0.023 to 0.036) | 0.64 |
| INL area (mm^2^) | 0.019 (-1.0 to 1.0) | 0.97 |
| Outer retina area (mm^2^) | 0.29 (0.055 to 0.52) | 0.017 |
| Drugs injected (aflibercept) | 0.2 (0.033 to 0.37) | 0.021 |

**Supplementary Table S3.** Relationships between best-corrected visual acuity at the 12-Month visit and variables obtained at the initial visit. INL, inner nuclear layer; logMAR, logarithm of the minimum angle of resolution; CI, confidence interval; No. = number.
